# Gastrointestinal Henoch–Schönlein purpura successfully treated with Mycophenolate Mofetil: Description of 2 case reports

**DOI:** 10.1097/MD.0000000000024093

**Published:** 2021-01-08

**Authors:** Maria Francesca Gicchino, Dario Iafusco, Maria Maddalena Marrapodi, Rosa Melone, Giovanna Cuomo, Angela Zanfardino, Emanuele Miraglia del Giudice, Alma Nunzia Olivieri

**Affiliations:** a Department of Woman, Child and General and Specialized Surgery; b Department of Precision Medicine, University of the Study of Campania “Luigi Vanvitelli,” Naples, Italy.

**Keywords:** abdominal pain, gastrointestinal bleeding, mycophenolate mofetile, purpura, Schönlein–Henoch syndrome

## Abstract

**Rationale:**

Henoch–Schönlein Purpura (HSP) is an acute small vessel vasculitis. It is the most common vasculitis in children. In majority of the cases, the disease is self-limited. Relapses can occur, in particular during the first year of the disease. There is no consensus on a specific treatment. The efficacy and safety of steroidal treatment in treating HSP is still controversial. Immunosuppressive treatment of HSP nephritis is used in patients with severe renal involvement (nephrotic range proteinuria and/or progressive renal impairment). The literature on immunosuppressive treatment of severe HSP without kidney involvement is scanty.

**Patients concerns:**

We report 2 case reports of 2 adolescents affected from Henoch–Schönlein Purpura and severe gastrointestinal involvement. Both patients presented a poor response to steroids treatment.

**Diagnoses:**

The diagnosis of HSP was made according to the diagnostic criteria published by European League against Rheumatism and Pediatric Rheumatology European Society in 2006

**Interventions:**

In consideration of the recurrence of the Henoch Schönlein Purpura and the gastrointestinal involvement, we decided to start Mycophenolate Mofetil treatment.

**Outcomes:**

In both patients all clinical manifestations resolved in few days.

**Lessons:**

In our cases of HSP with gastrointestinal involvement Mycophenolate Mofetil treatment has been very effective. This experience teaches us that immunosuppressive agents may be very useful to induce and maintain remission not only in renal involvement, but in all cases of persistent, recurrent, or complicated Henoch Schönlein Purpura in children.

## 1 Introduction

Henoch–Schönlein Purpura (HSP) is the most common systemic vasculitis in children.^[1]^ The majority of patients are under 10 years old.^[2]^ The annual incidence of HSP in children is estimated to be 15/100,000 cases.^[3]^ The proportion of males and females in children is close to 2:1.^[4]^ Diagnostic criteria were published by the European League against Rheumatism and the Pediatric Rheumatology European Society. They include palpable purpura in combination with at least one of the following manifestations: gastrointestinal involvement, immunoglobulin A deposition in biopsy, arthritis or arthralgia, and renal involvement^[5]^ (Table 1). Clinical manifestations are: palpable purpura (96%), arthralgia/arthritis (64%), abdominal pain (66%), gastrointestinal bleeding (28%), renal involvement (39%), subcutaneous edema (42%), orchitis (5%)_._ Patients rarely show severe pulmonary hemorrhage (1%), or cerebral vasculitis (2%).^[6]^ Purpuric rash is the most typical manifestation. Although recurrence is common among children (recurrence rate, 2.7%–66.2%), HSP prognosis is generally good. Significant morbidity and mortality are associated with gastrointestinal tract lesions and nephritis.^[7]^ The development of renal disease within the first 6 months after disease's onset or recurrent relapses associated with nephropathy suggest a poor prognosis for renal function.^[7]^ Additional poor prognosis factors are gastrointestinal bleeding, decreased factor XIII activity, hypertension, renal failure at onset.^[8]^ Treatment is supportive, with maintenance of good hydration, and with control of pain with analgesics. However, the efficacy and safety of the therapy with steroids and immunosuppressant drugs in treating HSP is still controversial,^[9,10]^ there are retrospective studies and case reports that suggest effective use of corticosteroids for the treatment of abdominal pain, renal involvement, and severe orchitis.^[11]^ Immunosuppressive treatment of HSP nephritis is used in patients with severe renal involvement (nephrotic range proteinuria and/or progressive renal impairment).^[12]^ The literature on immunosuppressive treatment of severe HSP without kidney involvement is scanty.^[13]^ In this article we report the successful use of MMF in 2 patients affected by HSP with gastrointestinal involvement.

**Table 1:** Modified from Ozen et al.

	Definition
Purpura (mandatory)	Purpura palpable or petechiae, in particular on lower limbs, not related to thrombocytopenia
At last 1 of the following	
Abdominal pain	Diffuse, acute coliky pain. May due to intussusception and gastrointestinal bleeding
Histopathology	Leucocytoclastic vasculitis with IgA deposit, or proliferative glomerulonephritis with IgA deposit
Arthritis or arthralgia	Arthritis or Arthralgia
Renal involvement	Proteinuria>0.3 g/24 h; spot urine albumin to creatinine ratio>30 mmol/mg, or 2+ on dipstickHematuria, red cell casts. Urine sediment showing >5 red cells per high power field or red cells casts

## 2 Case reports

### 2.1 Case number 1

At the age of 8 years, after a streptococcal pharyngitis treated with amoxicilline, patient presented pain in her lower limbs, difficult to walk and purpuric lesions to the limbs. During hospitalization in a local hospital she was treated with oral prednisone, 2 mg/kg/die. She was discharged for disappearance of symptoms after 1 week with diagnosis of suspected vasculitis and therapy with prednisone (2 mg/kg/die) for 10 days.

After 3 years of good conditions, patient presented hematemesis and purpuric lesions so she was hospitalized again. Laboratory tests were unremarkable for: complete blood count, kidney and liver parameters, electrolytes and coagulation test, antinuclear antibodies (ANA) celiac screening. Throat swab was negative. Urinalysis did not show neither proteinuria nor hematuria. Occult blood was present in the stool. Abdomen ultrasound was normal. Methylprednisolone in vein (2 mg/kg/die for 3 days) was administered, then oral prednisone (2 mg/kg/die) was prescribed. She was discharged after 10 days with Schonlein Henoch Vasculitis diagnosis. Oral prednisone was suspended after 10 days.

Four years later patient presented again purpuric lesions and hematemesis, so she was hospitalized. Patient underwent both to an esophagogastroduodenoscopy (EGDS) that was negative and to a skin biopsy, suggestive of leukocytoclastic vasculitis with IgA deposition, typical of HSP. HSP diagnosis was confirmed and oral prednisone (2 mg/kg/die, gradually tapered in a month) was prescribed, with an initial improvement of symptoms. At steroids reduction purpuric lesions, hematemesis, abdominal pain, and diarrhea came back again, so patient came to Rheumatology Unit of our Department for the first time. On the examination she presented diffuse purpuric lesions and abdominal pain. No arthritis was detected. Her blood tests were unremarkable for: complete blood count, liver and kidney function, ANA, extractable nuclear antigens (ENA), Anti neuthophil Cytoplasmic antibodies (ANCA), rheumatoid factor (RF), anti-double-stranded DNA (dsDNA), Anti-streptolysin O (ASO), immunoglobulins, complement C3 and C4. Factor XIII activity was reduced (65%, normal value > 75%). Inflammatory parameters were slightly increased: erythrocyte sedimentation rate (ERS) 25 mm/h (normal value <15 mm/h), C-reactive protein (CRP) 1 mg/dL (normal value < 0.8 mg/dL). Urinalysis was normal. Occult blood in the stool was present. Throat swab was negative. Abdomen ultrasound showed bowel wall thickening. Cardiac ultrasound was normal. Therapy with oral prednisone 25 mg in the morning and 20 mg in the evening was prescribed, with initial improvement of symptoms, but after a few days she presented new purpuric lesions on knees and feet, and abdominal pain. Due to the recurrence of her symptoms and the lack of a sustained response to steroids, we decided to start MMF treatment. Patient was treated with MMF (600 mg/m^2^ twice a day) for a month, in the same period of time prednisone was gradually reduced. All clinical manifestations resolved in 2 weeks. After the first month of treatment, MMF was gradually reduced for a period of 6 months with a complete regression of vasculitic lesions and abdominal symptoms. There was no evidence of relapse in a 6 months follow-up.

### 2.2 Case number 2

After an upper respiratory tract infection, a 13-year-old boy presented purpuric lesions on upper and lower limbs with spontaneous resolution in 3 days. After a week patient presented again purpuric lesions on the limbs and trunk associated with abdominal pain so he was brought to emergency department. The abdominal ultrasound was normal and urinalysis revealed mild proteinuria, but no treatment was prescribed. Due to the persistence of purpuric lesions, abdominal pain, and the onset of vomiting and gastrointestinal bleeding, the patient was hospitalized to our Department. On examination purpuric lesions on limbs and trunk were detected, patient referred both abdominal and testis pain. No signs of arthritis were found. Blood tests were unremarkable for: complete blood count, liver and kidney function, ERS, CRP, ANA, ENA, ANCA, RF, dsDNA, ASO, immunoglobulins, complement C3 and C4. Factor XIII activity was reduced (55%, normal value > 75%). Virological screening revealed Immunoglobulin M versus Influenza Virus. Urinalysis showed mild proteinuria. Throat swab was negative. Abdomen ultrasound showed bowel wall thickening, while testis ultrasound was normal. HSP diagnosis was confirmed. Methylprednisolone iv (2 mg/kg/die) was administered for 3 days, then oral prednisone (2 mg/kg/die) was prescribed, with resolution of symptoms. After a few days patient presented new purpuric lesions at the limbs and trunk and abdominal pain. Considering the recurrence of symptoms, the abdominal involvement, and the lack of a sustained response to steroids, MMF (600 mg/m^2^/d) was added to the therapy, and prednisone was gradually reduced over the course of month. Symptoms improved few days after starting to treat the patient with MMF. MMF treatment was continued for a month, and gradually reduced over 6 months, with regression of both purpuric lesions and abdominal pain. There was no evidence of relapse in a 6 months follow-up.

## 3 Discussion

We described 2 cases of patients affected by recurrent HSP with gastrointestinal involvement successfully treated with MMF. Gastrointestinal involvement is one of the most debilitating symptoms of HSP, which may prompt symptoms ranging from abdominal pain, vomiting, intussusception to gastrointestinal hemorrhage.^[14]^ Our patients presented abdominal pain and gastrointestinal bleeding. It has been reported that some patients with steroid-resistant gastrointestinal involvement may benefit from immunosuppressive treatment such as cyclophosphamide or MMF.^[14,15]^ MMF suppressing lymphocyte proliferation is very effectiveness in many autoimmune disorders even at pediatric age, such as lupus erythematous systemic.^[16,17]^ Recent studies suggest that mycophenolatic acid (MPA), the active metabolite of MMF, can inhibit the adhesion of leukocytes to endothelial cells, which is a key process in the pathogenesis of ANCA vasculitis.^[18]^ Therefore, MMF is used in children with HSP nephritic and nephrotic syndrome to achieve remission of proteinuria.^[19]^ Nikibakhsh et al^[13]^ in a case series reported 6 children suffering from HSP with gastrointestinal and renal involvement who failed to respond to systemic steroids, whereas MMF promptly treated the complications of the disease. Martin et al^[14]^ described a case of a child with severe gastrointestinal HSP, successfully treated with MMF. Wang et al^[20]^ in a recent observational study reported the effective use of MMF in 18 patients affected by HSP with gastrointestinal involvement unresponsive to systemic steroids.

Our patients presented a severe gastrointestinal involvement partially responsive to high dose of steroids, so we decided to start an immunosuppressive treatment with MMF. Adverse effects of MMF are less severe than other immunosuppressive drugs or steroids^[14,21]^ (Table 2). In children a long exposure to steroids could induce also growth impairment.^[21]^ After the beginning of MMF both vasculitis lesions and abdominal symptoms disappeared in few days. Neither of our patients developed side effects due to MMF. According to literature data^[13,14,20]^ and to our experience MMF seems to be a safe and effective medication to treat HSP. This drug could be used in case of steroids side effects, steroids dependency (2 relapses during steroid tapering), or steroids inefficacy.^[13]^ There are no official guidelines about how long MMF should be administered in HSP with gastrointestinal involvement. However, following a recent study, we administered MMF for 1 month, and then reduced its dosage gradually over the course of 6 months.^[13,20]^

**Table 2:** Side effects and dose of main drugs used for the chronic treatment of vasculitis.

	Cyclophosphamide (CYC)	Azathioprine	Mycophenolate mofetil (MMF)	Ciclosporin	Methotrexate	Corticosteroids (prednisone/metilprednisolone)
Dose	2–3 mg/kg once a day Per Os (PO) 2–3 mo; 0.5–1.0 g/m^2^ in vein (IV) monthly with mesna to prevent cystitis	0.5–2.5 mg/kg once a day PO for 1 y or more	(600 mg/m^2^ twice a day)	3–5 mg/kg/d PO in 2 divided doses	10–15 mg/m^2^/wk PO or SC (single dose)	1–2 mg/kgPO/IVIn case of bolus:MetilPrednisolone30 mg/kg max 1 g EV
Side Effects	Leucopenia; hemorrhagic cystitis; leukemia, lymphoma, transitional cell carcinoma of bladder	GI toxicity; hepatotoxicity; no conclusive data for cancer risk in children	Bone marrow suppression; severe diarrhoae; pulmonary fibrosis	Renal impairment, hypertension, hepatotoxicity, tremor, gingival hyperplasia, hypertricosis, lymphoma	Bone marrow suppression and interstitial pneumonitis, reversible elevation of transaminases, hepatic fibrosis	Growth impairmentDiabetesHypertensionDyselectrolitesEdemaHypokaliemiaOsteopeniaMusles atrophy

## 4 Conclusions

The report of these 2 cases suggests that MMF may be a promising therapeutic alternative to induce and maintain remission of recurrent HSP with gastrointestinal involvement. Our patients did not have adverse events associated with MMF therapy. Further research and multicenter clinical trials with long-term follow-up to confirm the efficacy of MMF in the treatment of HSP with gastrointestinal involvement are warranted to consolidate information about the effectiveness of MMF in treating HSP with gastrointestinal involvement.

## Acknowledgments

The authors are grateful to Maria Rosaria Taddeo and to Ranjith Kothalawalage for the English revision of this paper. Both of them give permission to be named.

## Author contributions

GMF conceived the paper, involvement in the diagnosis and follow-up of patient, analyzed and interpreted the patient data and first writer of paper; MMM, MR, and ZA diagnosis and management of patient, analyzed and interpreted the patient data, writer of paper and revision of bibliography; ID, CG, MDGE, and OAN supervision of the medical procedures, including the decision of the use of MMF, writer of paper.

All authors read and approved the final manuscript.

**Conceptualization:** Maria Francesca Gicchino, Dario Iafusco, Giovanna Cuomo, Angela Zanfardino, Emanuele Miraglia del Giudice, Alma Nunzia Olivieri.

**Data curation:** Maria Francesca Gicchino, Maria Maddalena Marrapodi, Rosa Melone, Giovanna Cuomo, Alma Nunzia Olivieri.

**Formal analysis:** Maria Francesca Gicchino, Maria Maddalena Marrapodi, Rosa Melone, Alma Nunzia Olivieri.

**Funding acquisition:** Maria Francesca Gicchino, Emanuele Miraglia del Giudice.

**Investigation:** Maria Francesca Gicchino, Dario Iafusco, Giovanna Cuomo, Angela Zanfardino, Emanuele Miraglia del Giudice, Alma Nunzia Olivieri.

**Methodology:** Maria Francesca Gicchino, Dario Iafusco, Maria Maddalena Marrapodi, Rosa Melone, Angela Zanfardino, Emanuele Miraglia del Giudice, Alma Nunzia Olivieri.

**Project administration:** Maria Francesca Gicchino, Emanuele Miraglia del Giudice, Alma Nunzia Olivieri.

**Resources:** Maria Francesca Gicchino, Emanuele Miraglia del Giudice, Alma Nunzia Olivieri.

**Software:** Maria Francesca Gicchino, Alma Nunzia Olivieri.

**Supervision:** Maria Francesca Gicchino, Dario Iafusco, Giovanna Cuomo, Angela Zanfardino, Emanuele Miraglia del Giudice, Alma Nunzia Olivieri.

**Validation:** Maria Francesca Gicchino, Maria Maddalena Marrapodi, Emanuele Miraglia del Giudice, Alma Nunzia Olivieri.

**Visualization:** Maria Francesca Gicchino, Maria Maddalena Marrapodi, Emanuele Miraglia del Giudice, Alma Nunzia Olivieri.

**Writing – original draft:** Maria Francesca Gicchino, Dario Iafusco, Maria Maddalena Marrapodi, Rosa Melone, Giovanna Cuomo, Angela Zanfardino, Emanuele Miraglia del Giudice, Alma Nunzia Olivieri.

**Writing – review & editing:** Maria Francesca Gicchino, Dario Iafusco, Maria Maddalena Marrapodi, Rosa Melone, Giovanna Cuomo, Angela Zanfardino, Emanuele Miraglia del Giudice, Alma Nunzia Olivieri.
